# Organizational contextual stimuli and health belief among migrant workers with pneumoconiosis: the mediating role of perceived health status

**DOI:** 10.3389/fpubh.2026.1788392

**Published:** 2026-04-24

**Authors:** Xingbang Chen, Jinsong Cai

**Affiliations:** School of Humanities and Social Science (School of Public Administration), Beihang University, Beijing, China

**Keywords:** health belief, migrant workers with pneumoconiosis, organizational contextual stimuli, perceived health status, structural equation modeling

## Abstract

**Background:**

Pneumoconiosis is the most prevalent occupational disease in China, with migrant workers being its primary victims. However, migrant workers with pneumoconiosis generally exhibit low health belief, which is associated with a decline in their quality of life. Organizational contextual stimuli are an important variable associated with the health belief of these workers. Perceived health status also plays a crucial role in this process. This study examines the associations among health belief, organizational contextual stimuli, and perceived health status among migrant workers with pneumoconiosis. It also explores factors associated with their health belief.

**Methods:**

This study is based on 1,109 valid samples completed by migrant workers with pneumoconiosis across eight Chinese provinces. It employed one-way analysis of variance (ANOVA) and independent samples *t*-tests to investigate differences in health belief and associated factors among these workers across various sociodemographic characteristics. Additionally, structural equation modeling (SEM) was utilized to examine the associative pathways linking organizational contextual stimuli, health belief, and perceived health status among these workers.

**Results:**

Results from one-way ANOVA and independent samples t-tests indicate differences in health belief and associated factors among migrant workers with pneumoconiosis across all sociodemographic characteristics except gender. SEM analysis reveals that organizational contextual stimuli are significantly associated with health belief among these workers (*β* = 0.287, *p* < 0.001) and perceived health status (*β* = 0.267, *p* < 0.001). Perceived health status is also significantly associated with health belief (*β* = 0.379, *p* < 0.001). Furthermore, perceived health status plays a statistically significant but modest mediating role in the relationship between organizational contextual stimuli and health belief among this population (indirect effect = 0.056, 95% CI: 0.041–0.096). The model exhibited acceptable fit (CMIN/DF = 1.972, RMSEA = 0.048, CFI = 0.931).

**Conclusion:**

It is suggested to strengthen organizational support and protection, enhance the health atmosphere and emotional well-being of migrant workers with pneumoconiosis, and cultivate health initiative among this population. In turn, this is associated with increased endogenous motivation for health belief in this population, is continuously associated with the strengthening of health belief, and improves the construction of health services for these workers.

## 1 Introduction

With the ongoing implementation of the “Healthy China 2030” Planning Outline and the Healthy China Initiative (2019–2030), China is intensifying its occupational health protection efforts to safeguard the physical well-being of all workers and promote safe and healthy socioeconomic development. Currently, the situation regarding the prevention and control of occupational diseases in China, particularly pneumoconiosis, remains complex and severe. According to the latest statistics released by China’s National Health Commission, 12,087 new cases of occupational diseases were reported nationwide in 2023. Among these, 8,051 new cases of occupational pneumoconiosis were reported, accounting for approximately 66.6% of the total (1). Pneumoconiosis is a systemic disease caused by prolonged inhalation of production-related mineral dust during occupational activities or in living environments, leading to diffuse nodular or reticular fibrosis of lung tissue. In severe cases, it can result in respiratory failure and death (2). Pneumoconiosis accounts for approximately 90% of all reported occupational diseases in China and remains incurable (3). Most patients are rural migrant workers, with estimates exceeding six million—more than 10 times the official number (4, 5).

Faced with the severe challenge of preventing and treating pneumoconiosis, relevant health authorities including the National Health Commission of China and member units of the Inter-Ministerial Joint Conference on Occupational Disease Prevention have successively issued a series of policy documents to advance pneumoconiosis control. These include the Action Plan for the Campaign to Tackle Pneumoconiosis, and the National Occupational Disease Prevention and Control Plan (2021–2025). These measures aim to deepen the campaign against pneumoconiosis, implement treatment protocols and strengthen prevention at the source. They also aim to ensure medical care and assistance for affected individuals—particularly migrant workers with pneumoconiosis. Additionally, the “Proposal of the Central Committee of the Communist Party of China on Formulating the 15th Five-Year Plan for National Economic and Social Development,” adopted at the Fourth Plenary Session of the 20th CPC Central Committee, also calls for “establishing and improving occupational injury protection systems”, “enhancing the social assistance system”, and “strengthening mental health and psychiatric services” (6).

However, most migrant workers with pneumoconiosis in China are constrained by their educational background, which is associated with generally low levels of health awareness, health skills and health status (7). Compounding this, some dust-exposed workplaces pay insufficient attention to dust-related pneumoconiosis prevention education and awareness campaigns. Given the disease’s relatively long latency period, most migrant workers remain unaware of their condition. Weak health protection during the early stages of illness, coupled with the absence of employment contracts or other documentation later on, often prevents occupational disease diagnosis. Consequently, they cannot access workers’ compensation benefits or relevant medical assistance. Poor access to healthcare services, weak family support, and inadequate social assistance create organizational stressors that increase psychological burden and are continuously associated with reduced levels of health belief among these workers. This hinders the cultivation of health literacy (8). Consequently, the quality of life for these workers lags behind, increasing their susceptibility to health risks (9) and posing challenges to China’s safe and healthy socioeconomic development. Therefore, it is critical to deconstruct the multidimensional layers of health belief among migrant workers with pneumoconiosis and to reveal the associative pathways between organizational contextual stimuli and their health belief. Exploring pathways associated with health belief is essential for sustaining pneumoconiosis prevention and control efforts. This, in turn, is crucial for curbing the disease’s high incidence, safeguarding the occupational health of the Chinese people, and promoting national economic development and social stability.

## 2 Literature review and hypotheses

### 2.1 Literature review

Health belief refers to an individual’s conceptual framework regarding health protection, disease prevention, and ensuring optimal quality of life. Specifically, it denotes the health-related convictions an individual holds, which are associated with health-promoting behaviors (10, 11). Health belief among migrant workers with pneumoconiosis refers to the perceptions, judgments, and cognitive constructs regarding their health status and related health behaviors that are associated with the experience of pneumoconiosis. Key dimensions include perceptions of susceptibility to pneumoconiosis, perceptions of its severity, perceptions of health benefits, perceptions of health barriers, self-efficacy, and action cues (12). This dimensional framework aligns with the overall structure of the health belief model (HBM) (13, 14).

Currently, most academic research on migrant workers’ health belief regarding pneumoconiosis has analyzed associated factors or proposed strategies from perspectives such as health-related quality of life, health awareness capacity, life hardships, pneumoconiosis prevention and treatment, and health protection and intervention. These studies primarily focus on two levels: organizational contextual stimuli and perceived health status, as well as individual characteristics (12). First, at the organizational contextual stimuli level, relevant studies in Western developed countries indicate that factors such as poor working conditions, weakened dissemination of medical knowledge (15), urban–rural development imbalance (5), health rights dilemmas (16), and lack of health services and medical insurance (17) may be correlated with reduced quality of life for migrant workers with pneumoconiosis. In severe cases, these factors may be linked to survival crises and social identity discrimination, which are further associated with the cultivation of health belief and health awareness among these workers. Chinese studies indicate that factors such as household economic pressure, charitable medical assistance (18), healthcare and labor protection conditions (19), social support (20), and environmental and relational quality (21) are significantly associated with the living conditions of migrant workers with pneumoconiosis. These factors also have a correlation with their health management awareness and capabilities. Second, at the level of perceived health status and individual characteristics, existing research indicates that there is a correlation between individual characteristics such as the medical history of pneumoconiosis among migrant workers (22), physical pathological responses (23), and educational levels (24) of migrant workers with pneumoconiosis and health belief. Building upon the HBM, related studies have found that factors including age at onset, duration of dust exposure, and occupation types (25) are associated with the self-management capabilities of these workers. Additionally, perceived health status factors such as physiological and psychological well-being, psychological dependence, and emotional experiences (21) are also associated with the health awareness and health behaviors of these workers.

In summary, existing academic literature has conducted fruitful research on the health belief of migrant workers with pneumoconiosis. However, most studies have indirectly deconstructed and analyzed the associated factors and mechanisms of the health belief from perspectives such as group quality of life and external environment. Empirical research on the mechanisms associated with the health belief of this population remains scarce. Furthermore, existing studies predominantly employ normative analysis while lacking quantitative analysis, rendering the feasibility and practicality of proposed countermeasures and recommendations subject to further exploration.

Therefore, this study builds upon the HBM and the “Stimulus-Organism-Response” (SOR) model. It draws from the theoretical framework previously established in this research, which encompasses organizational contextual stimuli, perceived health status, and socio-demographic characteristics to model the mechanisms associated with health belief among migrant workers with pneumoconiosis (12). Employing an empirical analysis paradigm, this study further examines variations in health belief and associated factors among this population across different individual characteristics. It clarifies the associative pathways and intensity of organizational contextual stimuli on health belief, as well as the mediating role of perceived health status. This addresses gaps in existing research, thereby providing theoretical insights and empirical data for pneumoconiosis prevention, rehabilitation studies, and health intervention practices among this population.

### 2.2 Theoretical basis

#### 2.2.1 Health belief model

**Figure 1:**
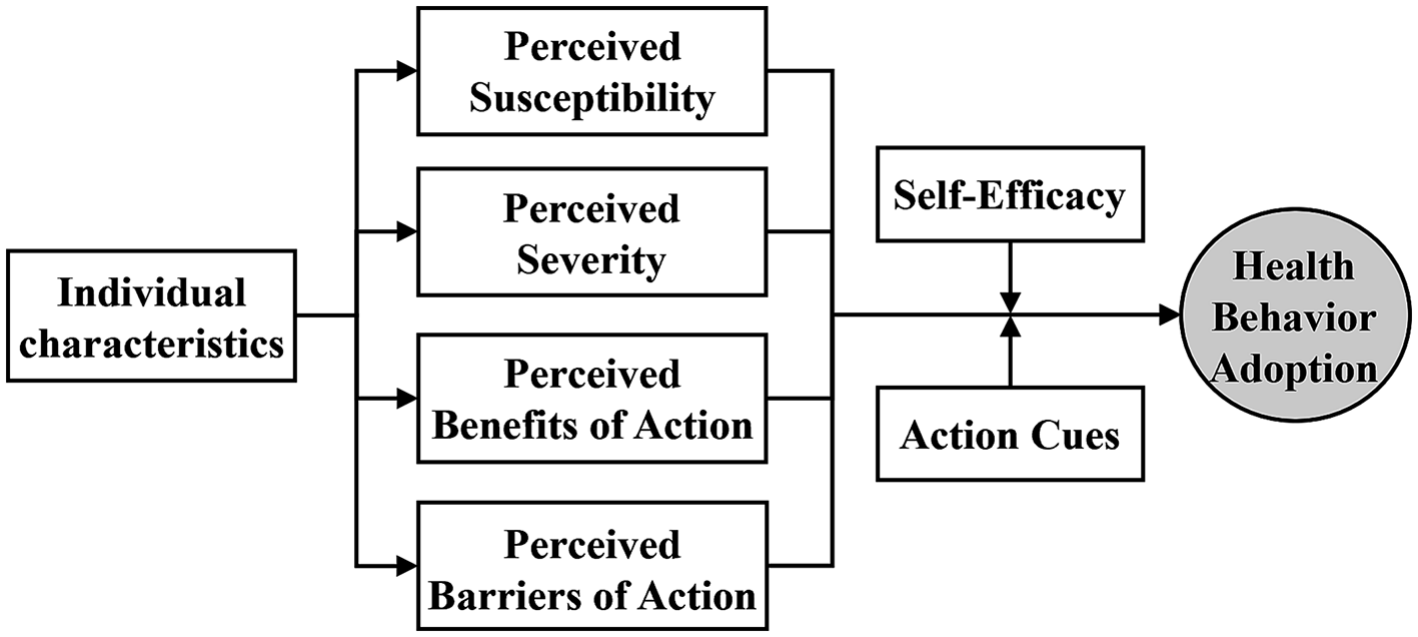
Health belief model.

The HBM was first proposed by psychologist Hochbaum in 1958 and initially applied to research exploring the relationship between individual behavior and health belief (26). Subsequently refined by American social psychologists such as Becker and Rosenstock, it gradually evolved into a significant theoretical framework capable of rationally explaining why people adopt health behaviors (13, 14). This model integrates social psychological theories including expectancy theory and cognitive theory. It focuses on individuals’ perceived threat of disease and their acceptance of health behaviors (7, 27), and is now widely applied in public health. The model structure comprises six core elements (Figure 1): Perceived Susceptibility, Perceived Severity, Perceived Benefits of Action, Perceived Barriers of Action, Self-Efficacy, and Action Cues. Perceived Susceptibility refers to an individual’s subjective perception and cognitive assessment of the probability of contracting a specific disease or exhibiting related symptoms; Perceived Severity refers to an individual’s subjective feelings and cognitive assessment of the consequences and harmfulness of a disease; Perceived Benefits of Action refers to an individual’s feelings, experiences, and emotions regarding the benefits gained from adopting a certain health behavior; Perceived Barriers of Action refers to an individual’s subjective assessment of potential obstacles such as difficulties, costs, or lack of ability when adopting a certain health behavior; Self-Efficacy refers to an individual’s subjective perception and cognitive assessment of their belief, ability, and initiative to adopt a health behavior and achieve favorable outcomes; Action Cues refer to internal or external motivating factors for an individual to adopt a health behavior, such as personal characteristics or work environment.

Focusing on the group of migrant workers with pneumoconiosis, individuals of different ages, years of service, occupations, and educational levels exhibit varying degrees of susceptibility awareness and severity perception regarding the probability, causes, and harmfulness of the disease. They also demonstrate differing levels of benefit recognition and barrier perception concerning the gains or costs associated with protecting their own health. Building upon this foundational understanding, self-efficacy centered on self-management and self-motivation emerges. Action cues, primarily rooted in physical capabilities and organizational health environments, also serve as essential prerequisites for these workers to cultivate health belief and adopt healthy behaviors.

#### 2.2.2 SOR model

**Figure 2:**
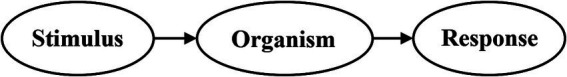
Stimulus-organism-response model.

SOR, also known as the “stimulus-organism-response” theoretical model, originates from psychology. First proposed by Mehrabian and Russell (28) in 1974 within environmental psychology research, it primarily explores the relationship between external environmental stimuli and individual consciousness and related behaviors. Woodworth (29) later incorporated subjective psychological factors into this model, emphasizing the role of individual cognition, emotions, personality, attitudes, and motivations in mediating the interaction between external stimuli and individual responses. The SOR model primarily consists of stimulus variables, mediating variables, and response variables (Figure 2). Mediating variables reflect the relationship between environmental stimuli and responses such as belief and behaviors (30). Specifically, it includes: External Environmental Stimuli (Stimulus), Organismic Cognition or Emotion (Organism), and Individual Response (Response). Among these, external environmental stimuli refer to factors that can trigger or induce changes in an individual’s internal or external states; organismic cognition or emotion refers to the intermediary psychological states—such as cognition, emotion—generated when an individual receives external environmental stimuli; Individual response refers to the subsequent psychological or behavioral reactions generated by the individual based on established cognition or emotion after being stimulated by external environmental stimuli. It is worth noting that the SOR model primarily highlights the mediating link between external environmental stimuli and individual responses. It reflects the rich cognitive thoughts and emotional states generated by individuals when confronting external environmental stimuli. This process alters psychological or behavioral patterns (31). This underscores the crucial role of the organism’s individual cognition and emotion.

Preliminary research indicates that the process of health belief formation among migrant workers with pneumoconiosis aligns precisely with the core logic of the SOR model (12). Specifically, these migrant workers develop corresponding perceptions of health environments in response to organizational contextual stimuli such as government health regulations, corporate health management, healthcare services, family economic conditions, and social assistance programs. These perceptions, coupled with physical pathological reactions, are associated with individual health awareness, health-related emotions, and perceived health status. Influenced by sociodemographic characteristics, these factors are in turn related to health belief.

The HBM and SOR model provide the theoretical foundation for this study. Specifically, the SOR model serves as the overarching analytical framework. Within the external environmental stimulus dimension, it encompasses organizational contextual stimuli generated by governments, enterprises, medical institutions, families, and society toward migrant workers with pneumoconiosis. Within the organismic cognition or affective dimension, it includes the overall perception factors of these workers regarding their own health status, which are associated with organizational contextual stimuli. At the individual response dimension, drawing upon the HBM, the study deconstructs the health belief constructs of migrant workers with pneumoconiosis. These include susceptibility to pneumoconiosis, perceived severity of the disease, perceived health benefits, perceived barriers to health, self-efficacy and action cues, which are related to the organizational contextual stimuli and perceived health status. That is, organizational contextual stimuli are correlated with migrant workers’ health belief, with perceived health status serving as a potential mediator. This theoretical framework further supports the formulation of the research hypotheses.

The application of the HBM or the SOR model in isolation is insufficient for a comprehensive understanding of the health belief of migrant workers with pneumoconiosis. Existing studies applying HBM or SOR independently have produced fragmented understanding. Although the HBM provides a detailed account of individual cognitive mediators of health behavior, it pays comparatively limited and unsystematic attention to the macro-environmental and organizational antecedents that shape these perceptions (7, 25). Conversely, while the SOR model effectively explicates the structural linkage between external stimuli and behavioral responses, it often lacks conceptual specificity regarding the internal psychological processes within the “Organism” component that translate stimuli into concrete actions (32). These theoretical gaps constrain the explanatory power of each model when applied independently to structurally vulnerable populations. No prior study has integrated these frameworks to model how organizational contextual stimuli shape health belief through perceived health status in vulnerable occupational populations.

To address these limitations, this study integrates the two frameworks. Specifically, it conceptualizes Organizational Contextual Stimuli (S), derived from the SOR model, as critical external antecedents that activate Perceived Health Status (O), which subsequently is associated with the core HBM constructs—perceived susceptibility, perceived severity, perceived benefits, perceived barriers, self-efficacy, and cues to action (R). This integrative framework connects structural-environmental factors associated with individual-level cognitive mechanisms, contributing to greater theoretical coherence. Such integration is particularly warranted given the structural constraints faced by migrant workers with pneumoconiosis, whose health belief are formed under conditions of labor informality, limited access to medical services, and economic precarity—contexts insufficiently addressed in conventional HBM applications centered on institutionally supported populations. In this respect, the SOR model provides contextual sensitivity, whereas the HBM contributes health-specific cognitive precision, together offering a more comprehensive account of health belief formation in disadvantaged settings.

### 2.3 Hypothesis

#### 2.3.1 Organizational contextual stimuli

Migrant workers diagnosed with pneumoconiosis often face difficulties in accessing their rightful social health services and assistance due to the absence of responsible entities. They grapple with challenges in workers’ compensation, inadequate representation of their interests, and insufficient labor protections. In this context, organizational factors—including local government policy choices, capital operation logic, and the supportive organizational forces from various sectors of society—are collectively associated with the quality of life and health status of migrant workers with pneumoconiosis. These factors, in turn, are associated with the development of their health belief (19). Therefore, the organizational contextual stimuli in this study refers to the collective impact of formal or semi-formal institutions on the living and working environment of these workers, in which “organization” covers a wide range of physical and social structures (33), including government, dust-related employment enterprises, medical institutions, etc. Although the functions of government, enterprises, and medical institutions are different, empirical evidence shows that they are related to the health belief of these workers through similar ways. For example, relevant empirical studies have found that the health belief and social support of Chinese coal miners are related to government welfare policies, corporate safety investment, and medical insurance coverage. Each factor is associated with differences in health protection behavior (7, 16). In addition, the quality of life of pneumoconiosis patients is associated with access to multi-level support, including government assistance, employer compensation, and quality of medical institutions (34). This “perceived organizational support” structure (35) has been verified in different organizational environments.

Specifically, the government, enterprises, and medical institutions are the three primary entities shaping the health belief of migrant workers with pneumoconiosis. Among these, the government’s functions in health management, supervision, support, and standardization collectively form a series of health regulations. Enterprises’ health management practices—including health education campaigns, prioritization of worker health, investment in safeguards, institutional development, service capacity, comprehensive dust control measures, and corporate responsibility—are jointly associated with the effectiveness of their health management efforts for this population. Healthcare institutions primarily undertake health service functions, including health intervention services, psychological support, occupational disease prevention services, and ensuring accessibility to medical care. The broad participation of various social stakeholders—including government, enterprises, and healthcare institutions—along with the coordination and collaboration between charitable assistance and social welfare programs (18), is associated with a supportive health environment for families of migrant workers with pneumoconiosis and society at large. These are collectively associated with their perceived health status, which may be associated with the cultivation of health belief. Concurrently, social assistance safeguards such as medical aid and work injury insurance obtained by these workers are positively associated with their quality of life, as well as with higher perceived health status and the formation of health belief. Conversely, the economic burden from medical treatment and household expenses, coupled with inadequate living security, is linked to poorer family economic conditions. This, in turn, weakens the quality of life and perceived health status while being negatively associated with the development of health belief. Based on the above analysis, the following hypothesis is proposed:

*H1*: Organizational contextual stimuli are positively associated with health belief among migrant workers with pneumoconiosis.

*H2*: Organizational contextual stimuli are positively associated with perceived health status.

#### 2.3.2 Perceived health status

Perceived health status of migrant workers with pneumoconiosis is a complex process and a critical factor in shaping their health belief. The health environment experienced by these workers is largely associated with external organizational contextual stimuli. Simultaneously, the real-time changes in their pathological responses continuously adjust their dynamic perceptions of health rights and protective measures. Specifically, in the health-conscious environment that has been formed, these workers increasingly perceive the work atmosphere, social environment, and family support encompassing encouragement from coworkers, societal care, and family companionship. This is associated with higher levels of their self-directed health awareness (36) and may be correlated with an individual’s perception of health centered on the recognition of health rights and health protection. Health Rights Dimension: Focus on understanding rights-related content such as occupational disease diagnosis, work injury insurance, labor protection, health rights safeguarding, and rights exercise. Health Protection Dimension: Focus on understanding protective content such as health awareness, health investment, pneumoconiosis prevention, and self-health protection. At the same time, the pathological reactions experienced by migrant workers after falling ill also heighten their perception of personal health. For instance, when pneumoconiosis reaches a higher stage or symptoms become more severe, migrant workers develop a deeper awareness of the disease, often becoming more conscious of the importance of personal health protection and safeguarding their rights. Furthermore, upon recognizing their health status, these workers develop a vague sense of their overall well-being. This is associated with health-related emotions—including a sense of responsibility for health, a sense of belonging to health, and health-related affect—which are linked to more positive mental states and lower levels of negative emotions (36). This process facilitates a comprehensive subjective understanding of pneumoconiosis, cultivating positive perceptions of health benefits and health barriers. Consequently, it is positively associated with individuals’ motivation to adopt health protection measures (37). Based on the above analysis, the following hypothesis is proposed:

*H3*: Perceived health status is positively associated with health belief among migrant workers with pneumoconiosis.

*H4*: Perceived health status plays a partial mediating role in the statistical association between organizational contextual stimuli and health belief among migrant workers with pneumoconiosis.

#### 2.3.3 Sociodemographic characteristics variable

Existing research indicates that various sociodemographic variables are also associated with the health-related quality of life among migrant workers with pneumoconiosis, which are further associated with the cultivation and reinforcement of health belief (20, 38). For instance, these workers who differ in age, years of service, occupation types, number of dust-exposed workplaces, daily working hours, and educational levels exhibit varying subjective perceptions regarding the origins of pneumoconiosis, its associated hazards, and necessary protective measures. Consequently, their health belief and willingness to engage in health-protective behaviors also diverge. Based on field interviews and research findings, younger male migrant workers face greater household financial burdens, which are associated with a tendency to prioritize income over personal health and health belief cultivation (39, 40). Migrant workers with shorter tenure tend to recognize the dangers of pneumoconiosis earlier, which is associated with higher levels of health belief (41). Migrant workers engaged in occupations like mining and stone processing, who are exposed to high dust concentrations in their work environments and receive more dust-related health education from their employers, have a stronger awareness of their own health status (42, 43). The more dust-exposed workplaces a dust-related disease migrant worker has been employed in, the more work experience they gain, the more opportunities they have to learn health protection knowledge and skills, and the greater their awareness of the importance of preventing dust-related diseases, which is associated with higher levels of health belief (43). These workers who work shorter daily hours tend to have better perceived health status, stronger health awareness, and better self-health management capabilities (44). Migrant workers with higher educational levels demonstrate deeper understanding of dust protection and the hazards of pneumoconiosis, enabling them to more profoundly recognize the physical and mental harm caused by the disease and the health support provided by their external environment (8). Furthermore, the vast majority of Chinese migrant workers with pneumoconiosis are male. The small number of female workers generally aligns with their male counterparts in terms of perceived health status, health awareness, and self-management capabilities (39). Based on the above analysis, the following hypothesis is proposed:

*H5*: Differences exist in the levels of health belief and in associated factors among migrant workers with pneumoconiosis across other sociodemographic characteristics besides gender.

## 3 Research design and methods

### 3.1 Variable measurement

To ensure that the collected sample data accurately and effectively reflects the health belief characteristics of migrant workers with pneumoconiosis. This study, grounded in the practical context of health intervention practices for Chinese migrant workers with pneumoconiosis, drew upon established scales from relevant academic research fields (32, 45–56). Integrating theoretical features of the HBM and the SOR model, it comprehensively considered the organizational contextual stimuli, perceived health status, and health belief characteristics faced by these workers. Consequently, a measurement scale for key variables was designed and developed (Table 1), comprising 3 dimensions and 15 items.

**Table 1:** Primary variable measurement item scale.

Latent variable	Observed variable	Measurement item content
Organizational contextual stimuli	Government health regulations (A1)	Government agencies will proactively provide you with pneumoconiosis relief services.
	Corporate health management (A2)	When engaged in dust-related work, employers will ensure proper health protection against pneumoconiosis.
	Health services from medical institutions (A3)	Medical institutions will actively offer occupational disease diagnosis or examinations.
	Household financial status (A4)	Before and after your pneumoconiosis diagnosis, your family’s living standards are very poor.
	Social assistance and security (A5)	Current social assistance programs can meet most of your needs.
Perceived health status	Perceived health atmosphere (B1)	You can clearly sense the health-conscious atmosphere around you.
	Physical pathological responses (B2)	You distinctly feel the pain and discomfort following your pneumoconiosis diagnosis.
	Individual health perception (B3)	You consider prompt occupational disease diagnosis critically important.
	Health-related emotions (B4)	You view thorough pneumoconiosis rehabilitation treatment as both your duty and a necessity.
Health belief among migrant workers with pneumoconiosis	Perceived susceptibility to pneumoconiosis (C1)	You believe prolonged exposure to dusty environments increases susceptibility to pneumoconiosis.
	Perceived severity of pneumoconiosis (C2)	You perceive pneumoconiosis as causing significant suffering in your life.
	Perceived health benefits (C3)	You believe effective prevention and rehabilitation treatment for pneumoconiosis can positively be associated with your quality of life.
	Perceived health barriers (C4)	You find discussing pneumoconiosis prevention and rehabilitation treatment uncomfortable.
	Self-efficacy (C5)	You are highly motivated to pursue pneumoconiosis prevention and rehabilitation treatment.
	Action cues (C6)	You believe you have the capability to pursue pneumoconiosis prevention and rehabilitation treatment.

The questionnaire items of organizational contextual stimuli dimension draw on measurement indicators defined in relevant studies—such as organizational safety climate and social norms (32, 45). It is mainly adapted from Zohar and Luria’s (32) organizational climate scale and Schneider et al.’s (46) organizational climate framework, contextualized to Liu et al.’s (7) and Huang et al.’s (21) measures of migrant worker health environments in Chinese pneumoconiosis contexts. It focuses on five indicator dimensions: government health regulations, corporate health management, health services from medical institutions, household financial status, and social assistance security. These five dimensions in turn assess the respondents’ perceptions and evaluations of organizational contextual stimuli through the following questions: “Government agencies will proactively provide you with pneumoconiosis relief services.” “When engaged in dust-related work, employers will ensure proper health protection against pneumoconiosis.” “Medical institutions will actively offer occupational disease diagnosis or examinations.” “Before and after your pneumoconiosis diagnosis, your family’s living standards are very poor.” “Current social assistance programs can meet most of your needs.”

The questionnaire items of perceived health status dimension draw upon psychological and affective measurement factors defined in relevant studies, such as sense of security and responsibility (47, 48) and psychological belonging (49, 50). It is mainly developed based on Ware and Sherbourne’s (51) SF-36 health perception subscale, Keyes’s (52) emotional well-being dimensions, and Diener et al.’s (53) subjective well-being scales, informed by Qiu et al.’s (20) pneumoconiosis quality of life research. It focuses on four indicator dimensions: perceived health atmosphere, physical pathological responses, individual health perception, and health-related emotions. These four dimensions in turn assess the respondents’ perceptions and evaluations of perceived health status through the following questions: “You can clearly sense the health-conscious atmosphere around you.” “You distinctly feel the pain and discomfort following your pneumoconiosis diagnosis.” “You consider prompt occupational disease diagnosis critically important.” “You view thorough pneumoconiosis rehabilitation treatment as both your duty and a necessity.”

The questionnaire items of health belief dimension for migrant workers with pneumoconiosis draw upon established health belief scales widely applied in assessing miners’ unsafe behaviors and health self-management behaviors (54, 55). It is mainly adapted from Champion’s (56) HBM Scale, Rosenstock’s (13) and Becker’s (57) original HBM formulations, and Rosenstock et al.’s (58) social learning integration, with pneumoconiosis-specific adaptations drawing from Wu et al. (25) and the previous work of this study (12). It focuses on six indicator dimensions: perceived susceptibility to pneumoconiosis, perceived severity of pneumoconiosis, perceived health benefits, perceived health barriers, self-efficacy, and action cues. These six dimensions in turn assess the respondents’ perceptions and evaluations of health belief through the following questions: “You believe prolonged exposure to dusty environments increases susceptibility to pneumoconiosis.” “You perceive pneumoconiosis as causing significant suffering in your life.” “You believe effective prevention and rehabilitation treatment for pneumoconiosis can positively be associated with your quality of life.” “You find discussing pneumoconiosis prevention and rehabilitation treatment uncomfortable.” “You are highly motivated to pursue pneumoconiosis prevention and rehabilitation treatment.” “You believe you have the capability to pursue pneumoconiosis prevention and rehabilitation treatment.”

The scale employs a 5-point Likert scale for testing items related to primary variables (1 represents “strongly disagree,” 5 represents “strongly agree”). Additionally, seven items were designed to assess seven socio-demographic variables among migrant workers with pneumoconiosis: age, gender, years of service, occupation types, number of dust-exposed workplaces, daily working hours, and educational levels. Further refinement involved inviting eight experts in dust-related pneumoconiosis prevention, occupational health, and health belief studies to review and evaluate the items. Additionally, 20 migrant workers were consulted through interviews to ensure the variable definitions, measurement dimensions, and item content design were both thematically relevant and operationally sound.

### 3.2 Data sources and descriptive statistical analysis

When selecting survey samples, it is essential to ensure their representativeness and relevance. This entails choosing regions with a high concentration of migrant workers suffering from pneumoconiosis, while also encompassing diverse groups of affected workers—those exhibiting varying symptoms, different stages of pneumoconiosis, and distinct socio-demographic characteristics. Therefore, while ensuring the feasibility and practicality of data collection, the survey sample was defined across eight provinces: Shanxi, Qinghai, Sichuan, Hunan, Liaoning, Jiangxi, Gansu, and Henan. These provinces have relatively large populations of migrant workers with pneumoconiosis and numerous dust-exposed workplaces. The primary occupation is “mining,” with a high prevalence of high-dust work environments. Additionally, these provinces have relatively mature and effectively implemented specialized assistance policies for pneumoconiosis patients. This facilitates the collection of multi-dimensional and multi-level data on assistance policies, rehabilitation treatment, medical coverage, labor protections, and other relevant aspects concerning this population.

The research team members have repeatedly participated in field investigations conducted by the BD Public Welfare Foundation regarding the living conditions of Chinese migrant workers suffering from pneumoconiosis. The BD Public Welfare Foundation is a national public welfare organization dedicated to assisting China’s 6 million pneumoconiosis-affected farmers and committed to promoting prevention and ultimately eliminating the disease. It currently operates 174 volunteer teams across all provinces and regions, with over 17,000 registered volunteers. Over the past decade, the foundation has assisted more than 135,000 dust-pneumonia-affected farmers and their families across all 31 provinces, municipalities, and autonomous regions. Its efforts have directly contributed to the implementation of China’s Action Plan for the Prevention and Control of Dust Pneumonia and a series of related policies supporting the welfare of affected farmers.

This survey employed field research interviews and questionnaire distribution to gather empirical data for analysis. Research interviews and questionnaires targeted migrant workers with pneumoconiosis across the aforementioned eight provinces. To minimize potential recall and social desirability biases inherent in self-report questionnaires, several rigorous procedural controls were implemented during data collection. First, trained volunteers administered the questionnaires through face-to-face interviews using standardized instructions. This approach avoided leading questions and allowed clarification of items, thereby enhancing the accuracy of participants’ recollections relative to self-administered questionnaires. Second, all responses were collected anonymously and sealed immediately upon completion to safeguard confidentiality. All participants were informed that participation was voluntary and that they could withdraw at any time without consequence. Third, an informed consent form was provided with the questionnaire, explicitly stating that the data would be used solely for academic research and handled with strict confidentiality, thereby promoting honest reporting and mitigating social desirability bias. Given the vulnerability of migrant workers with pneumoconiosis, particular attention was paid to ensuring that no psychological pressure or coercion occurred during face-to-face interviews. Personal identifiers were not recorded, and all data were stored securely for research purposes only. Finally, two reverse-scored items (A4 and C4) were incorporated to evaluate response consistency.

To ensure a sufficient and valid sample size for stable and reliable results, the research team distributed 150 questionnaires in each of the above eight provinces through the BD Public Welfare Foundation in collaboration with relevant medical institutions, pneumoconiosis volunteer service stations, and rural health centers, totaling 1,200 questionnaires. One-on-one face-to-face interviews were conducted with 698 individuals, and one-to-many centralized face-to-face interviews were conducted with 502 people. The research interviews were conducted face-to-face with the respondents before they filled out the questionnaire. The interview explained the purpose of the study, the meaning of each item and the matters needing attention in filling out the questionnaire in detail. The interview purpose is to reduce the understanding deviation of the respondents with lower education level and improve the quality of the questionnaire data. Due to varying literacy levels among migrant workers with pneumoconiosis, trained investigators provided standardized guidance. When necessary, investigators read the questionnaire items aloud and recorded respondents’ verbal answers. Whenever possible, respondents completed the questionnaire independently.

To acknowledge respondents’ time and effort, valid participants received modest on-site incentives (e.g., masks, hand sanitizers, and basic pneumoconiosis care kits). High response rates were achieved through individualized guidance from trained researchers, collaboration with local medical institutions and volunteer stations, and respondents’ recognition of the importance of pneumoconiosis prevention research among migrant workers. Before distributing the questionnaires, the research objectives and precautions were thoroughly explained to respondents. After the questionnaires were collected, 91 forms with blank entries, obvious inconsistencies, or non-compliance were uniformly excluded (missing data rate: 7.58%; listwise deletion was applied). Missing data were assessed prior to analysis and found to be minimal. Given the low level of missingness, this approach is unlikely to have biased the results. 1,109 valid questionnaires were recovered, representing a response rate of 92.42%. The sample composition is shown in Table 2.

**Table 2:** Descriptive statistical analysis of demographic characteristics in the survey sample (sample size = 1,109).

Socio-demographic characteristics variable	Options (percentage, unit: %)	Options (percentage, unit: %)
Age	39 years old or below (0.59%)	40–49 years old (11.79%)
	50–59 years old (46.37%)	60 years old or above (41.26%)
Gender	Male (98.04%)	Female (1.96%)
Years of service	5 years or less (21.61%)	6–15 years (24.75%)
	16–25 years (21.22%)	26 years and above (32.42%)
Occupation types	Mining (88.21%)	Metal smelting (4.52%)
	Machine manufacturing (0.39%)	Construction (3.14%)
	Highway, railway, water conservancy, and hydropower construction (1.96%)	Stone processing (1.77%)
Number of dust-exposed workplaces	1 (35.36%)	2–4 (28.29%)
	5–9 (14.73%)	10 or more (21.61%)
Daily working hours	5 h or less (1.18%)	6–8 h (11.79%)
	9-10 h (46.95%)	11 h or more (37.72%)
	Irregular hours (2.36%)	
Educational levels	Primary school or below (61.69%)	Junior high school (32.61%)
	Technical secondary school (0.39%)	High school or higher (5.30%)

It should be noted that the interviews were used as an auxiliary research method for the survey, and the interview data were mainly used for the optimization of the questionnaire scale and the improvement of the quality of the questionnaire data, rather than for independent statistical analysis. The core empirical analysis of the study was based on the quantitative data of the questionnaire.

### 3.3 Statistical analysis

All statistical analyses were conducted using SPSS 27.0 and AMOS 29.0 software.

First, normality testing is a prerequisite for conducting scale data analysis. Currently, the maximum likelihood (ML) estimation method is widely used in SEM. However, employing this method necessitates ensuring that scale data passes normality testing. Normality tests were conducted using SPSS 27.0 on scale data, calculating the mean, standard deviation, skewness, and kurtosis for each item score under every variable to assess the normality of scale measurement items. Given that this scale employs a positive scoring model ranging from 1 to 5, an average score between 3 and 4 indicates that the sample group’s cognitive understanding and scoring behavior toward the measurement items generally fall at a moderately high level. As an indicator of data dispersion, smaller standard deviations suggest that while differences exist in the distribution of the sample across these variables, the degree of dispersion is low. Absolute values of skewness and kurtosis below 2 were considered indicative of approximate normality (59).

Second, reliability analysis was conducted on the valid sample data collected via the questionnaire using SPSS 27.0 software. Reliability was evaluated using Cronbach’s *α* coefficient, with values >0.7 indicating acceptable internal consistency.

Third, validity was assessed through exploratory factor analysis (EFA) and confirmatory factor analysis (CFA). For EFA, the Kaiser-Meyer-Olkin (KMO) measure (>0.7) and Bartlett’s test of sphericity (*p* < 0.05) were used to verify sampling adequacy; principal component analysis (PCA) with varimax rotation was applied, and factors with eigenvalues >1 were retained. PCA is a data reduction technique that explains total variance without assuming latent constructs (60). The use of PCA in this study was intended to simplify the observed variables and provide a preliminary empirical basis for subsequent modeling, rather than to establish latent constructs in a strict psychometric sense. PCA is widely used for initial scale exploration in public health research, and critically, the structure identified via PCA was subsequently validated using CFA (61). To assess potential common method variance (CMV), an unrotated PCA (Harman’s single-factor test) was conducted on all 15 measurement items. For CFA, ML estimation was used. Convergent validity was assessed by factor loadings (>0.5), average variance extracted (AVE > 0.5), and composite reliability (CR > 0.8). Discriminant validity was established when the square root of AVE for each latent variable exceeded its correlations with other latent variables (62).

Fourth, to compare health belief and associated factors across sociodemographic groups, independent samples *t*-tests (for gender) and one-way ANOVA (for age, years of service, occupation types, number of dust-exposed workplaces, daily working hours, and educational levels) were performed. Effect sizes were calculated as Cohen’s *d* for *t*-tests and eta-squared (*η*^2^) for ANOVA.

Fifth, SEM was employed to test the hypothesized pathways among organizational contextual stimuli, perceived health status, and health belief. SEM is a statistical analysis method that combines features of factor analysis and multiple regression analysis, enabling the handling of complex multivariate problems (63, 64). When performing model fitting analysis using ML, model fit was evaluated using the following criteria: Chi-Square/Degrees of Freedom Ratio (CMIN/DF) < 3, Goodness-of-Fit Index (GFI) > 0.9, Root Mean Square Residual (RMR) < 0.08, Standardized Root Mean Square Residual (SRMR) < 0.08, Comparative Fit Index (CFI) > 0.9, Normed Fit Index (NFI) > 0.9, Non-Normative Fit Index (NNFI) > 0.9, and Root Mean Square Error of Approximation (RMSEA) < 0.08 (65). Based on the theoretical model and research hypotheses, the structural equation model depicting the associative mechanism of health belief on migrant workers with pneumoconiosis was constructed using AMOS 29.0 software. The significance of path coefficients was examined using critical ratios (C.R.) with a threshold of C.R. >1.96 (*p* < 0.05).

Finally, mediation analysis was conducted using the bootstrap method (66) with 5,000 resamples and a 95% confidence interval, supplemented by *Z*-value calculations via the coefficient multiplication method. The indirect effect was considered statistically significant if the bias-corrected and percentile confidence intervals excluded zero.

## 4 Results

### 4.1 Normality test

The results of the normality test are shown in Table 3. First, the mean scores for each measurement item are primarily concentrated between 3 and 4. This indicates that most respondents possess a relatively clear, positive, and in-depth understanding of the measurement items, enabling them to make relatively prepared and reasonable judgments based on their own health status, actual experiences, and life experiences. Second, the standard deviations for each measurement item fall within the narrow range of 1.163 to 1.786. This indicates that respondents’ cognitive understanding and evaluative opinions regarding most observed variables are relatively consistent, with no significant divergence or variation. This further supports the reliability of the scale data. Third, the absolute values of the skewness and kurtosis coefficients for each measurement item were all less than 2. This indicates that the vast majority of respondents’ scores were close to the mean, with relatively few deviating significantly from it. This suggests that the scale data generally followed a normal distribution (59), meeting the research requirements for SEM analysis.

**Table 3:** Results of normality test for measurement items.

Variable	Item	Minimum	Maximum	Mean	Standard deviation	Skewness	Kernel
Organizational contextual stimuli	A1	1	5	3.15	1.707	0.629	−0.395
	A2	1	5	3.27	1.163	0.362	0.053
	A3	1	5	3.57	1.579	−0.424	−0.405
	A4	1	5	3.88	1.401	−1.701	0.543
	A5	1	5	3.21	1.763	0.185	−0.765
Perceived health status	B1	1	5	3.15	1.607	0.629	−1.395
	B2	1	5	3.50	1.786	−1.055	−0.239
	B3	1	5	3.30	1.423	0.531	−1.051
	B4	1	5	3.01	1.236	0.781	−0.317
Health belief among migrant workers with pneumoconiosis	C1	1	5	3.08	1.712	0.085	−1.748
	C2	1	5	3.06	1.263	0.072	−1.159
	C3	1	5	3.14	1.482	0.065	−1.357
	C4	1	5	3.98	1.512	−1.106	−0.483
	C5	1	5	3.10	1.422	0.542	−1.031
	C6	1	5	3.30	1.235	0.781	−0.317

### 4.2 Reliability and validity test

#### 4.2.1 Reliability test

The overall Cronbach’s *α* coefficient for the scale was 0.905. Specifically, the coefficients for organizational contextual stimuli (0.860), perceived health status (0.831), and health belief among migrant workers with pneumoconiosis (0.842) all exceeded 0.8 (Table 4). This indicates that the scale exhibits high reliability in terms of both item-data consistency and overall stability, demonstrating good stability and consistency.

**Table 4:** Reliability test results.

Dimension	Number of items	Cronbach’s *α*
Organizational contextual stimuli	5	0.860
Perceived health status	4	0.831
Health belief among migrant workers with pneumoconiosis	6	0.842
Overall	15	0.905

#### 4.2.2 Validity test

The EFA results showed a KMO value of 0.857, exceeding the threshold of 0.7. The Bartlett’s sphericity test yielded an observed value of 1014.621 with a significance level of 0.000, below the 0.05 threshold. The resulting scree plot revealed three initial components with eigenvalues exceeding 1.0. The extracted components reflect underlying patterns in the observed data; however, they should not be interpreted as true latent constructs in the strict sense of common factor models. While PCA components are not strictly equivalent to latent factors, this structure was subsequently validated by CFA. The rotated loadings’ sum of squares indicated a cumulative variance explained of 74.310%, exceeding 60%, suggesting good item representativeness and high overall explanatory power. The factor component matrix after orthogonal rotation showed that all observed variables corresponding to items fell within their respective latent variable categories, indicating high construct validity of the scale.

The results of Harman’s single-factor test indicate that the first factor accounted for 32.67% of the total variance, well below the 40% threshold, indicating that CMV was not a serious concern and did not pose a substantive threat to inferences about the relationships among variables.

Table 5 shows that the factor loadings for organizational contextual stimuli, perceived health status, and health belief among migrant workers with pneumoconiosis all exceed 0.5, indicating strong representativeness of the items corresponding to each latent variable. AVE values all exceed 0.5, demonstrating that the latent variables adequately explain the observed variables. CR values all exceed 0.8, indicating high internal consistency within the constructs. Thus, the model exhibits good convergent validity.

**Table 5:** Convergent validity test results.

Latent variable	Observed variable	Standardized loading	CR	AVE
Organizational contextual stimuli	A1	0.721	0.862	0.686
	A2	0.761		
	A3	0.734		
	A4	0.795		
	A5	0.715		
Perceived health status	B1	0.780	0.845	0.706
	B2	0.812		
	B3	0.710		
	B4	0.734		
Health belief among migrant workers with pneumoconiosis	C1	0.764	0.911	0.754
	C2	0.812		
	C3	0.721		
	C4	0.790		
	C5	0.845		
	C6	0.833		

Table 6 shows that the AVE square root of each latent variable (located on the main diagonal of the table) was all greater than the correlation coefficients between that latent variable and other latent variables. This indicates that the internal consistency of each latent variable exceeded the correlations between latent variables, meaning all dimensions of the scale passed the rigorous test of discriminant validity. Therefore, a structural equation model can be constructed based on the scale (62).

**Table 6:** Distinctiveness of validity test results.

	AVE	Health belief among migrant workers with pneumoconiosis	Organizational contextual stimuli	Perceived health status
Health belief among migrant workers with pneumoconiosis	0.754	**0.868**		
Organizational contextual stimuli	0.686	0.875	**0.828**	
Perceived health status	0.706	0.793	0.821	**0.840**

The bold diagonal values represent the square roots of AVE; the values below the diagonal denote the correlation coefficients between latent variables and other latent variables.

### 4.3 Differential test

The differential test results presented in Table 7:

**Table 7:** Results of the test for differences in scores across dimensions of the scale among migrant workers with different categories of pneumoconiosis.

Category differences		Mean ± standard deviation		
		Organizational contextual stimuli	Perceived health status	Health belief among migrant workers with pneumoconiosis
Age	39 years old or below	2.90 ± 0.53	2.58 ± 0.52	2.84 ± 1.07
	40–49 years old	3.40 ± 0.64	3.42 ± 0.80	3.71 ± 1.03
	50–59 years old	3.41 ± 0.76	3.82 ± 0.79	3.24 ± 1.10
	60 years old or above	3.52 ± 0.79	3.36 ± 0.76	3.00 ± 1.09
	*F*	1.746	1.373	8.938
	*p*	0.157	0.250	0.000***
	*η* ^2^	0.005	0.004	0.024
Gender	Male	3.28 ± 0.76	3.39 ± 0.78	2.95 ± 1.11
	Female	3.31 ± 0.68	3.09 ± 0.81	3.41 ± 0.93
	*F*	−0.533	1.226	−1.284
	*p*	0.595	0.221	0.200
	Cohen’s *d*	0.040	0.390	0.430
Years of service	5 years or less	3.22 ± 0.61	2.98 ± 0.69	3.49 ± 1.05
	6–15 years	2.99 ± 0.69	3.02 ± 0.77	2.96 ± 1.19
	16–25 years	3.34 ± 0.78	3.38 ± 0.81	3.18 ± 1.13
	26 years and above	3.55 ± 0.80	3.44 ± 0.74	3.20 ± 1.04
	*F*	14.396	12.890	4.584
	*p*	0.000***	0.000***	0.004**
	*η* ^2^	0.038	0.034	0.012
Occupation types	Mining	3.52 ± 0.77	3.35 ± 0.78	3.32 ± 1.12
	Metal smelting	3.22 ± 0.60	2.84 ± 0.47	3.36 ± 0.94
	Machine manufacturing	2.80 ± 0.42	2.90 ± 0.35	3.23 ± 1.89
	Construction	3.21 ± 0.60	3.04 ± 0.85	3.18 ± 1.23
	Highway, railway, water conservancy, and hydropower construction	3.60 ± 0.81	3.50 ± 0.97	3.08 ± 1.29
	Stone processing	3.41 ± 0.80	3.59 ± 0.91	2.99 ± 0.93
	*F*	1.541	2.818	0.467
	*p*	0.163	0.010**	0.833
	*η* ^2^	0.007	0.013	0.002
Number of dust-exposed workplaces	1	3.18 ± 0.67	2.92 ± 0.71	3.10 ± 1.14
	2–4	3.15 ± 0.81	3.14 ± 0.81	3.16 ± 1.15
	5–9	3.43 ± 0.78	3.41 ± 0.68	3.26 ± 1.12
	10 or more	3.31 ± 0.80	3.39 ± 0.80	3.23 ± 1.02
	*F*	2.101	13.087	5.527
	*p*	0.050*	0.000***	0.000***
	*η* ^2^	0.006	0.034	0.015
Daily working hours	5 h or less	3.17 ± 0.76	3.40 ± 0.61	3.39 ± 0.87
	6–8 h	3.46 ± 0.67	3.36 ± 0.82	3.29 ± 1.12
	9-10 h	3.27 ± 0.77	3.30 ± 0.78	3.15 ± 1.14
	11 h or more	3.29 ± 0.77	3.06 ± 0.75	3.05 ± 1.08
	Irregular hours	3.08 ± 0.71	3.07 ± 0.93	3.09 ± 1.05
	*F*	1.055	2.023	3.132
	*p*	0.378	0.041*	0.000***
	*η* ^2^	0.004	0.007	0.011
Educational levels	Primary school or below	2.99 ± 0.78	2.87 ± 0.76	3.07 ± 1.11
	Junior high school	2.96 ± 0.72	3.00 ± 0.80	3.08 ± 1.11
	Technical secondary school	3.10 ± 1.56	3.13 ± 1.59	3.09 ± 2.36
	High school or higher	3.19 ± 0.74	3.10 ± 0.79	3.16 ± 1.05
	*F*	0.684	1.660	1.697
	*p*	0.002**	0.000***	0.000***
	*η* ^2^	0.002	0.004	0.005

***p < 0.001; **p < 0.01; *p < 0.05.

First, the *p*-value for age in the health belief dimension among migrant workers with pneumoconiosis was less than 0.05, with a small effect size (*η*^2^ = 0.024), indicating differences in health belief levels across different age groups, but the difference is small. Specifically, migrant workers aged 40–49 exhibited the highest health belief levels, those aged 50–59 showed moderately high levels, those aged 60 and above demonstrated moderate levels, while those aged 39 and below had the lowest levels.

Second, there was no significant gender difference in the levels of health belief and associated factors among this population. The *p*-values for all dimensions exceeded 0.05, Cohen’s *d* values were all below 0.10, suggesting negligible effects and overall similarity between male and female migrant workers with pneumoconiosis in health belief, organizational contextual stimuli, and perceived health status.

Third, the *p*-values for years of service were all less than 0.05 across the three dimensions of health belief, organizational contextual stimuli, and perceived health status, with small sizes (*η*^2^ = 0.038 for organizational contextual stimuli, 0.034 for perceived health status, 0.012 for health belief). This indicates differences in these three dimensions among migrant workers with pneumoconiosis across different years of service levels, but the differences were small. Specifically, these workers who had worked for 5 years or less exhibited the highest levels of health belief. However, overall, those with longer years of service scored higher on organizational contextual stimuli and perceived health status.

Fourth, the *p*-value for occupation types in the perceived health status dimension is less than 0.05, with a small effect size (*η*^2^ = 0.013), indicating differences in perceived health status among migrant workers with pneumoconiosis across different occupation types, but the difference is small. Specifically, these workers engaged in mining, stone processing, and highway/railway/water conservancy/hydropower construction reported moderately high levels of perceived health status, while those in construction, machinery manufacturing, and metal smelting reported moderately low levels.

Fifth, the *p*-value for the number of dust-exposed workplaces was less than 0.05 across all three dimensions—health belief, organizational contextual stimuli, and perceived health status, with small sizes (*η*^2^ = 0.006 for organizational contextual stimuli, 0.034 for perceived health status, 0.015 for health belief), indicating differences among migrant workers with pneumoconiosis based on the number of dust-exposed workplaces in these dimensions, but the differences were small. Specifically, these workers who had worked in more dust-exposed workplaces scored higher on organizational contextual stimuli, perceived health status, and health belief. Those with 5–9 dust-exposed workplaces scored highest across these dimensions, while those with 10 or more dust-exposed workplaces scored slightly lower than those with 5–9 workplaces.

Sixth, the *p*-values for daily working hours were both less than 0.05 in the dimensions of health belief and perceived health status among migrant workers with pneumoconiosis, with small sizes (*η*^2^ = 0.007 for perceived health status, 0.011 for health belief), indicating differences in these dimensions across groups with varying daily working hours, but the differences were small. Specifically, migrant workers with shorter daily working hours scored higher in both health belief and perceived health status dimensions.

Seventh, the *p*-value for educational levels was less than 0.05 across all three dimensions—health belief, organizational contextual stimuli, and perceived health status—among this population, with small sizes (*η*^2^ = 0.002 for organizational contextual stimuli, 0.004 for perceived health status, 0.005 for health belief). This indicates differences in these dimensions among workers with varying educational levels, but the differences were small. Specifically, migrant workers with higher educational levels scored higher on organizational contextual stimuli, perceived health status, and health belief.

The results indicate that although the difference tests reached statistical significance—which may be related to the large sample size—the corresponding effect sizes were small (*η*^2^ = 0.002–0.038; Cohen’s *d* < 0.10). This suggests that, while Hypothesis H5 is statistically supported, the observed differences are of limited practical significance. The magnitude of these effects is minimal, suggesting that organizational contextual stimuli, perceived health status, and health belief may be more strongly associated.

### 4.4 SEM analysis and hypothesis test

**Figure 3:**
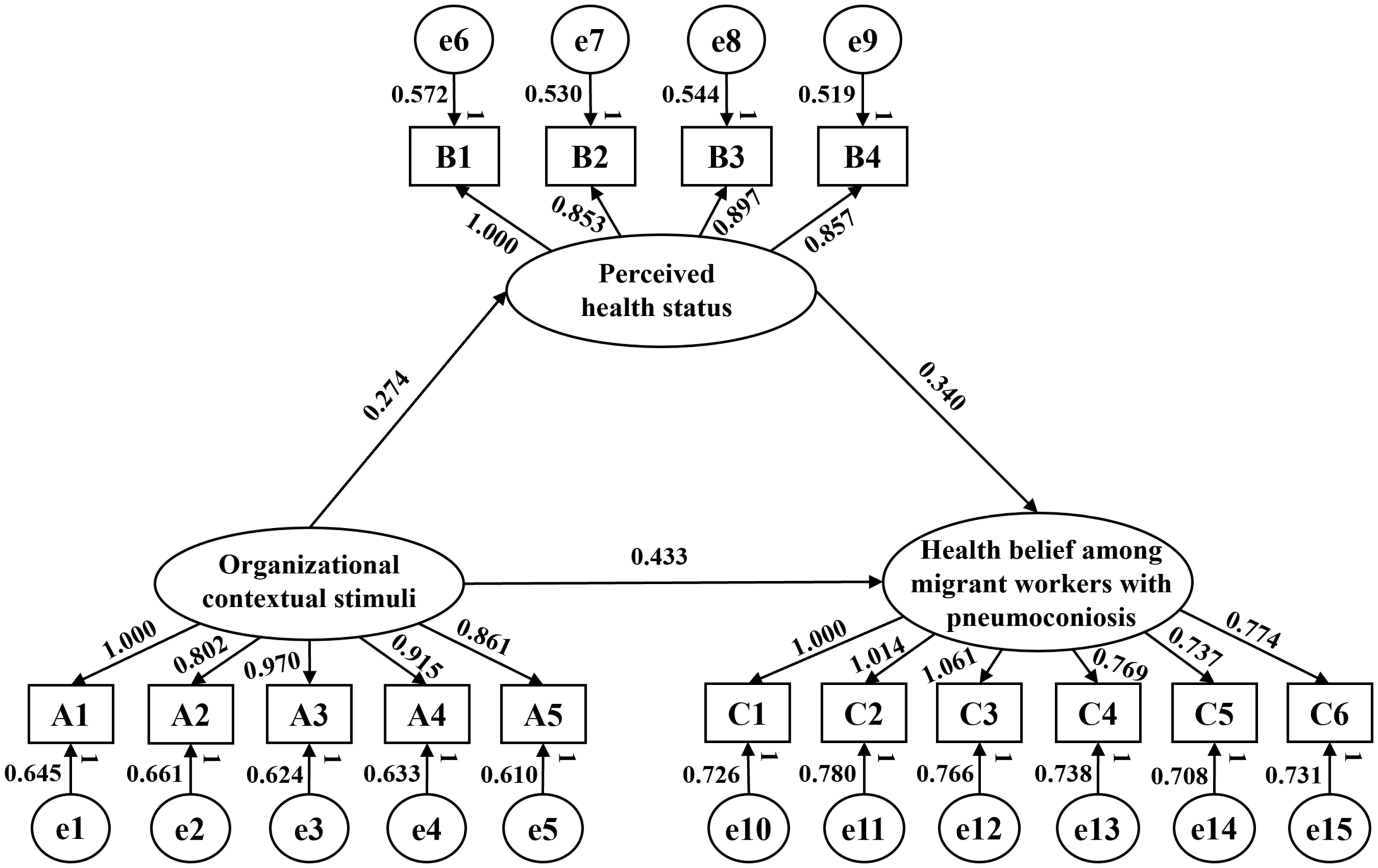
Structural equation model of the mechanism associated with health belief among migrant workers with pneumoconiosis.

The structural equation model of the mechanism associated with health belief among migrant workers with pneumoconiosis comprises 3 latent variables, 15 observed variables, and 3 paths. The SEM analysis results are presented in Figure 3.

The model fit test results are presented in Table 8: All fit indices met the recommended thresholds (CMIN/DF = 1.972 < 3, GFI = 0.924 > 0.9, RMR = 0.052 < 0.08, SRMR = 0.050 < 0.08, CFI = 0.931 > 0.9, NFI = 0.920 > 0.9, NNFI = 0.913 > 0.9, and RMSEA = 0.048 < 0.08), indicating acceptable model fit, and the predicted results show high consistency with actual conditions. Therefore, no further modifications are required for the model, and it can be used to validate the research hypotheses.

**Table 8:** Model fit index test results.

Fit indices	Evaluation reference criteria (adaptation values)	Test results	Compatibility
CMIN/DF	>1.0 and <3.0	1.972	Yes
GFI	>0.9, the closer to 1 the better the fit	0.924	Yes
RMR	<0.05 indicates excellent fit; <0.08 indicates good fit	0.052	Yes
SRMR	<0.05 indicates excellent fit; <0.08 indicates good fit	0.050	Yes
CFI	>0.9, the closer to 1 the better the fit	0.931	Yes
NFI	>0.9, the closer to 1 the better the fit	0.920	Yes
NNFI	>0.9, the closer to 1 the better the fit	0.913	Yes
RMSEA	<0.05 indicates excellent fit; <0.08 indicates good fit	0.048	Yes

Table 9 shows that the absolute values of the C.R. for all three hypothesized paths exceed 1.96, with path coefficients demonstrating statistical significance. Thus, hypotheses H1, H2, and H3 are all validated.

**Table 9:** Path coefficients and hypothesis test results.

Path	UnStd.	Std.	S.E.	C.R.	*p*	Hypothesis
Organizational contextual stimuli → health belief among migrant workers with pneumoconiosis	0.433	0.287	0.114	7.674	***	H1√
Organizational contextual stimuli → perceived health status	0.274	0.267	0.015	6.021	***	H2√
Perceived health status → health belief among migrant workers with pneumoconiosis	0.340	0.379	0.179	6.542	***	H3√

*** indicates significance at *p* < 0.001.

### 4.5 Mediation effect test

In the mediation effect test results (Table 10), the total effect TE’s confidence interval does not include zero, and the *Z*-value is 8.124 (>1.96), indicating a possible indirect effect. Both the confidence intervals for the direct effect (DE) and the indirect effect (IE) excluded zero, with *Z*-values of 7.325 and 4.589 (both >1.96), respectively. Therefore, the partial mediating role was observed. Specifically, perceived health status played a partial mediating role in the pathway from organizational contextual stimuli to health belief among migrant workers with pneumoconiosis. The mediating effect was 0.056, providing support for Hypothesis H4, though the effect is modest.

**Table 10:** Results of the mediational effect test.

Effect type	UnStd.	Product of coefficients		Bootstrapping			
		SE	*Z*	Bias-corrected 95% CI		Percentile 95% CI	
				Lower	Upper	Lower	Upper
Direct effect	0.433***	0.036	7.325	0.321	0.417	0.322	0.417
Indirect effect	0.056***	0.012	4.589	0.042	0.097	0.041	0.096
Total effect	0.489***	0.037	8.124	0.344	0.476	0.343	0.475

*** indicates significance at the 1% confidence level.

## 5 Discussion

### 5.1 Key findings and theoretical contributions

This study focuses on the association between organizational contextual stimuli and the health belief of migrant workers with pneumoconiosis. Empirical analysis indicates that organizational contextual stimuli are significantly and positively associated with the health belief of these workers, consistent with prior research findings (17, 21, 36). However, this study further reveals that organizational contextual stimuli also positively associated with migrant workers’ overall perceived health status, which is further positively correlated with health belief. Specifically, the mediation model confirms that perceived health status partially mediates the relationship between organizational contextual stimuli and health belief among these workers. It is important to note that while statistically significant, the mediating effect (*β* = 0.056) accounts for approximately 11.5% of the total effect. This suggests that perceived health status is a meaningful but not dominant mediator in this relationship. The main correlation path of health belief is still the direct association with organizational contextual stimuli. This nuanced finding aligns with research indicating that for this population, external structural support (e.g., policies, compensation) is often more strongly associated with their outlook than internal psychological states, which can be constrained by severe disease burden and socioeconomic challenges (37, 40). By systematically examining the mediating pathways of perceived health status, this study elucidates the potential underlying association between organizational contextual stimuli and health belief among migrant workers with pneumoconiosis, thereby deepening our understanding of their health belief and the mechanisms that shape it.

The core theoretical contribution of this study lies in the innovative integration and contextual adaptation of the HBM and the SOR model, thereby overcoming the limitations associated with the isolated application of either framework in existing occupational health research. First, this study systematically links organizational contextual stimuli of the SOR model with the micro-level cognitive structures in the HBM, addressing prior research that has either overlooked the correlation of organizational contextual stimuli or failed to provide a nuanced analysis of individual cognition. Second, the abstract construct of the “organism” in the SOR framework is refined into a measurable perceived health status, while the broad notion of “response” is refined into a six-dimensional health belief structure grounded in the HBM. Furthermore, the study empirically demonstrates the partial mediating role of perceived health status in the relationship between organizational contextual stimuli and health belief, clarifies the internal associational pathway between organizational situational stimuli and health belief, and supplements the key theoretical links missing in the single application of HBM or SOR models in existing research.

### 5.2 Further discussion

First, organizational contextual stimuli are positively associated with the health belief of migrant workers with pneumoconiosis, consistent with prior research (17, 21, 36, 37, 40). However, it also triggers critical reflection compared to the broader international literature. While studies from high-income countries have similarly documented the associations between workplace and policy environments and occupational disease patients’ health behaviors (40), the strength of these associations and the specific pathways observed here may reflect China’s unique institutional context. For instance, studies conducted in coal-mining populations in Brazil and Europe have similarly demonstrated that institutional protection and access to occupational health services significantly shape workers’ health-related quality of life and behavioral intentions (22, 23). In the model of this study, organizational contextual stimuli—particularly government health regulations and corporate management—emerged as key associative factors, suggesting that in China’s centralized policy environment, top-down institutional support may be especially influential. It resonates with research from Thailand (67) and Africa (68), where informal workers similarly lack legal protections and social security coverage. Unlike contexts characterized by more institutionalized compensation systems, the Chinese migrant worker population operates within a fragmented labor protection structure, which may amplify the salience of organizational contextual stimuli in shaping perceived health status.

Secondly, organizational contextual stimuli are indirectly related to the development of stable health belief among migrant workers with pneumoconiosis, with positive perceived health status associated with this relationship. The analytical framework and measurement paradigm constructed based on the SOR model and HBM demonstrate (12) that favorable external organizational contextual stimuli help these workers establish a systematic understanding and deep awareness of their health condition, thereby being associated with the activation of endogenous motivation for generating health belief. The indirect effect (0.056) accounts for 11.45% of the total association, falling in the small-to-moderate range (66, 69). Its practical significance could be interpreted within the specific context of pneumoconiosis prevention among migrant workers. The modest indirect effect and cross-sectional design caution against interpreting this as a strong causal mechanism. This pattern is consistent with mediation effects commonly observed in social and behavioral research, where statistically significant but modest indirect effects reflect the complexity of underlying processes. Following recent recommendations in population health research (70), small effect sizes can be associated with notable differences at the population level in contexts where interventions are implemented at scale. Given that over six million migrant workers suffer from pneumoconiosis in China, even a small mediation effect translates into meaningful health improvements for a substantial number of individuals. Moreover, given the chronic and incurable nature of pneumoconiosis, even modest improvements in health belief can accumulate over time, contributing to better self-management and quality of life (7, 37). These associations are observed primarily in relation to government health management and supervision, employer-led dust prevention, and medical psychological support—which are associated with strengthened perceptions of a supportive health environment. These organizational contextual stimuli are associated with awareness of health rights, self-protection, health-related emotions, and the reinforcement of core health belief, including perceived susceptibility, severity, benefits, barriers, self-efficacy, and cues to action.

Thirdly, the modest mediation effect necessitates considering explanations beyond the tested SOR sequence. Bidirectional relationships may exist: drawing on longitudinal studies of health and social capital (71), higher health belief may be associated with better perceived health status, potentially through increased health-seeking behaviors and self-management. For instance, migrant workers with stronger self-efficacy (a dimension of health belief) may be more likely to utilize available health services, thereby improving their perceived health environment and physical well-being. Second, unmeasured psychological constructs—such as institutional trust, perceived injustice, or health literacy—may mediate or moderate the observed relationships (5, 24). Organizational contextual stimuli may be associated with lower levels of stigma and higher collective identity among sufferers, and these factors are in turn associated with both perceived health status and health belief. Third, survivorship bias may partially explain the positive association between organizational contextual stimuli and perceived health status, as workers in more supportive environments may remain engaged in assistance networks. Future longitudinal research could further disentangle these potential mechanisms.

Additionally, this study found significant differences in the levels of health belief and associated factors among migrant workers with pneumoconiosis across various socio-demographic characteristics, excluding gender. Younger workers placed greater emphasis on work income while neglecting their own health, demonstrating insufficient awareness of the hazards of pneumoconiosis and consequently exhibiting relatively lower levels of health belief. Migrant workers with pneumoconiosis employed in mining, stone processing, and highway, railway, water conservancy, and hydropower construction—who were exposed long-term to dusty work environments—exhibited a stronger overall perceived health status (72). Migrant workers with shorter years of service, more dust-exposed employers, shorter daily working hours, and higher educational levels tend to have a more thorough understanding of the hazards of pneumoconiosis and the importance of prevention and treatment, which is associated with higher levels of health belief. Simultaneously, migrant workers with longer years of service, more dust-exposed employers, shorter daily working hours, and higher educational levels exhibit stronger perceptions regarding external organizational contextual stimuli and their own health status, consistent with previous research findings (24, 25, 34, 73).

These findings must be interpreted within China’s specific sociopolitical and healthcare context. First, the eight sampled provinces have relatively mature pneumoconiosis relief policies; in regions with weaker implementation, the correlation between organizational contextual stimuli and health belief may be attenuated. Second, China’s rapid urbanization and internal migration patterns mean that many affected workers lack stable urban residency, which is associated with reduced access to health services and labor protections. This institutional barrier may amplify the importance of organizational support. Third, cultural factors—such as the strong family orientation and collectivist values in rural China—may shape how perceived health status translates into health belief. For example, family economic pressure (A4 in the scale) may play a stronger associative role in this context than in individualistic societies. Cross-cultural comparative studies are needed to test whether the mediation mechanism observed here operates similarly in other countries with different healthcare systems and cultural norms.

### 5.3 Implications and suggestions for public health

The findings of this study provide several important implications for public health practice and policy, particularly in the context of occupational health protection for vulnerable populations such as migrant workers with pneumoconiosis. Based on the findings of this study, to enhance the effectiveness of health interventions for this population, which may be related to the strengthening of health belief, and to improve their health protection awareness and capabilities, the following policy implications are proposed:

First, strengthen organizational support and protection, and improve the construction of health services for migrant workers with pneumoconiosis. This study emphasizes the importance of optimizing and improving the material support and institutional guarantee of this population by revealing the relationship between organizational contextual stimuli and the health belief of this population. Based on the moderate effect size and cross-sectional design of this study, the pilot work of organizational support and improvement can be carried out in provinces with relatively mature pneumoconiosis relief policies. The government could focus on optimizing the basic pneumoconiosis relief and certification system, aiming to solve the problems of pneumoconiosis diagnosis difficulties and lack of labor contracts for migrant workers, and set up special small funds for one-stop medical assistance, rather than large-scale fundraising, so as to reduce the economic and psychological barriers for migrant workers to obtain organizational support, and carry out health rights education activities for migrant workers with low educational backgrounds. Dust-related enterprises may consider taking the leading role in reducing dust exposure by prioritizing the implementation of elimination, substitution and engineering control measures in line with the occupational disease prevention hierarchy of control. On this basis, enterprises would benefit from strengthening workplace health education activities, optimize the provision and standardized use guidance of personal protective equipment (PPE), and carry out targeted pneumoconiosis prevention and control training in dust-exposed workplaces with long operation time, to form a multi-level dust prevention and control system combining engineering management and individual protection. Medical institutions can introduce simplified pneumoconiosis health guidance services (such as free physical examination follow-up, short-term psychological counseling) for migrant workers with pneumoconiosis in the sample provinces and develop an easy-to-understand health manual suitable for their low education level.

Second, strengthen the health environment and emotional support for migrant workers with pneumoconiosis may be associated with higher levels of intrinsic motivation for health belief. The results of this study show that there is a partial mediating role of perceived health status. Although the association is not large, it indicates that emotional support intervention may be associated with moderate but potentially meaningful improvement. In view of the resource constraints of the rural environment in which most workers in China are located, volunteer doctors can be organized to carry out group psychological sessions for these workers by using the existing community or village health infrastructure, focusing on alleviating anxiety and other negative emotions caused by physical discomfort and guiding them to establish a simple sense of self-health responsibility. The community and the pneumoconiosis volunteer service station could be jointly constructed to establish a peer support network among pneumoconiosis patients who have recovered. Pneumoconiosis rehabilitation skills training and health literacy workshops could be implemented in stages to cultivate a targeted health awareness atmosphere and guide self-health management and active protection among these workers. This micro-intervention is consistent with the formation of health-related emotions and can continuously activate the mediating role of perceived health status.

Third, enhance the health agency of migrant workers with pneumoconiosis and continuously strengthen their health belief cognition. According to the heterogeneity results of socio-demographic characteristics, the health belief of pneumoconiosis migrant workers varied with age, service years, number of workplaces exposed to dust, daily working hours, and education level, but not by gender. Therefore, differentiated interventions could be designed to improve awareness of the susceptibility and severity of pneumoconiosis among migrant workers with low levels of health belief. For workers with longer working hours per day and lower health belief, institutional reform of dust-related workplace exposure time may be more strongly associated with health belief than health education at the individual level. For workers with a low education level, health communication could be intuitive, simple, and effective. In addition, the development of health literacy interventions targeting these workers’ self-efficacy and action cues is recommended, as these dimensions are most likely to change. Self-management seminars for pneumoconiosis symptom monitoring, as well as guidance and assistance for occupational disease diagnosis and compensation claims for these workers, can be carried out to improve the health self-efficacy and compliance of these workers and provide a strong guarantee for their health management services.

## 6 Conclusion

This study, grounded in the HBM and the SOR model, utilized 1,109 valid samples completed by migrant workers with pneumoconiosis across eight Chinese provinces. Employing SEM, one-way ANOVA, and independent samples t-tests, it conducted an in-depth investigation into the mechanisms associated with health belief among this population. The findings reveal the following conclusions:

First, organizational contextual stimuli are significantly and positively associated with the health belief of migrant workers with pneumoconiosis. When external organizational contextual stimuli are more intense, these workers are more likely to have higher health awareness and skills, which is associated with stronger health belief.

Second, perceived health status plays a partial mediating role between organizational contextual stimuli and health belief among migrant workers with pneumoconiosis, though the indirect effect is modest. Organizational contextual stimuli are positively associated with their perceived health status, which in turn is positively associated with their health belief. After receiving external organizational assistance such as health services and medical aid, these workers develop psychological feelings and emotional responses at the individual health protection level. These responses vary in type and degree. They are associated with health belief cognition regarding pneumoconiosis prevention.

Third, significant differences exist in the levels of health belief and associated factors among migrant workers with pneumoconiosis across other socio-demographic characteristics besides gender. Variations in subjective perceptions of pneumoconiosis among workers differing in age, years of service, occupation types, number of dust-exposed workplaces, daily working hours, and educational levels are correlated with disparities in their health belief levels, perceptions of external organizational contextual stimuli, and perceived health status.

### 6.1 Limitations

Although this study systematically examined the mechanism of the relationship between organizational contextual stimuli and health belief among migrant workers with pneumoconiosis through perceived health status, several areas warrant further refinement.

First, due to the large sample size, and the exclusive reliance on questionnaire-based data, some methodological bias may be present. Although procedural controls were implemented, subjective reporting errors cannot be entirely ruled out. To enhance the robustness of findings, future research could employ longitudinal tracking samples, incorporate objective data, and integrate observational or behavioral data for triangulation. This approach would explore the dynamic interactions among organizational contextual stimuli, perceived health status, and health belief among migrant workers with pneumoconiosis.

Second, the current sample primarily focused on provinces with large populations of migrant workers with pneumoconiosis and relatively mature pneumoconiosis relief policies. It did not consider provinces with large migrant worker populations but relatively underdeveloped relief policies, nor provinces with intermediate migrant worker populations and developing relief policies. Therefore, increasing sample size and geographic diversity could be considered to enhance the generalizability of the findings.

Third, the measurement model in this study was both developed and tested using the same sample. As such, cross-validation using an independent dataset was not performed. This may introduce a potential risk of overfitting and limit the generalizability of the findings. Future research could cross-validate the factor structure in an independent sample to further assess its robustness and external validity.

Fourth, the exploratory analysis relied on PCA. While this study used PCA for data reduction followed by CFA validation, it does not conform to the assumptions of common factor models and therefore may not fully capture latent psychological constructs. Future research could consider principal axis factoring or parallel analysis.

Last, this study adopts a cross-sectional research design, which can only reveal the associative relationships and theoretical mediating pathways among organizational contextual stimuli, perceived health status and health belief, but cannot empirically verify the causal relationship between variables. This limitation is particularly relevant given the modest magnitude of the indirect effect. Future longitudinal follow-up studies are needed to further validate the causal mechanism of the above variables.

## Data Availability

The raw data supporting the conclusions of this article will be made available by the authors, without undue reservation.
